# Systems resilience in the implementation of a large-scale suicide prevention intervention: a qualitative study using a multilevel theoretical approach

**DOI:** 10.1186/s12913-023-09769-x

**Published:** 2023-07-11

**Authors:** Louise A Ellis, Yvonne Zurynski, Janet C Long, Robyn Clay-Williams, Eline Ree, Mitchell Sarkies, Kate Churruca, Fiona Shand, Chiara Pomare, Maree Saba, Cecilie Haraldseid-Driftland, Jeffrey Braithwaite

**Affiliations:** 1 Centre for Healthcare Resilience and Implementation Science, Australian Institute of Health Innovation, Macquarie University, Level 6, 75 Talavera Road, North Ryde, NSW Australia; 2 Centre Faculty of Health Sciences, SHARE - Centre for Resilience in Healthcare, University of Stavanger, Stavanger, Norway; 3 Black Dog Institute, University of New South Wales, Sydney, Australia

**Keywords:** Resilience, Resilience in healthcare, Complexity, Suicide prevention, Implementation

## Abstract

**Background:**

Resilience, the capacity to adapt and respond to challenges and disturbances, is now considered fundamental to understanding how healthcare systems maintain required levels of performance across varying conditions. Limited research has examined healthcare resilience in the context of implementing healthcare improvement programs across multiple system levels, particularly within community-based mental health settings or systems. In this study, we explored resilient characteristics across varying system levels (individual, team, management) during the implementation of a large-scale community-based suicide prevention intervention.

**Methods:**

Semi-structured interviews (*n*=53) were conducted with coordinating teams from the four intervention regions and the central implementation management team. Data were audio-recorded, transcribed, and imported into NVivo for analysis. A thematic analysis of eight transcripts involving thirteen key personnel was conducted using a deductive approach to identify characteristics of resilience across multiple system levels and an inductive approach to uncover both impediments to, and strategies that supported, resilient performance during the implementation of the suicide prevention intervention.

**Results:**

Numerous impediments to resilient performance were identified (e.g., complexity of the intervention, and incompatible goals and priorities between system levels). Consistent with the adopted theoretical framework, indicators of resilient performance relating to anticipation, sensemaking, adaptation and tradeoffs were identified at multiple system levels. At each of the system levels, distinctive strategies were identified that promoted resilience. At the individual and team levels, several key strategies were used by the project coordinators to promote resilience, such as building relationships and networks and carefully prioritising available resources. At the management level, strategies included teambuilding, collaborative learning, building relationships with external stakeholders, monitoring progress and providing feedback. The results also suggested that resilience at one level can shape resilience at other levels in complex ways; most notably we identified that there can be a downside to resilience, with negative consequences including stress and burnout, among individuals enacting resilience.

**Conclusions:**

The importance of considering resilience from a multilevel systems perspective, as well as implications for theory and future research, are discussed.

**Supplementary Information:**

The online version contains supplementary material available at 10.1186/s12913-023-09769-x.

## Background

Over the past decade, resilient healthcare (RHC) has grown to underpin a new paradigm of safety [1–8]. Contemporary reviews of RHC have identified that to gain a better understanding of resilience, further research is needed to identify how resilience is distributed at different system levels [9–11]. Studies within RHC have predominantly focused on clinical microsystems at the ‘sharp end’ and how frontline healthcare professionals within hospital settings collectively adapt, ‘work around’, or enable things to go well [10, 12]. As a result, there is a need for multilevel studies that investigate how agents at various levels of the system create environmental and contextual conditions under which service providers work and perform in resilient ways [12], thereby narrowing the gap between conceptualisations of work-as-imagined (WAI) and work-as-done (WAD). There is also a need for RHC research to extend understanding outside of the hospital setting, into the broader context in which staff operate—in primary care, outpatient and community settings [9, 10], and specifically in mental health systems and settings [13].

Another noteworthy gap in the RHC literature is the limited discussion of how agents within the system may be personally affected by their efforts to maintain system resilience; such considerations appear to have been ignored, even “denied” [14]. The resilience required among individuals charged with effecting change during the planned implementation of system innovations, is seldom discussed. The time is ripe for this issue to be explored in the context of RHC, particularly in light of the COVID-19 pandemic, which has caused major disruptions across all system levels and created a need for rapid systems change and innovation, as well as ongoing adaptation by healthcare workers, resulting in widespread mental health issues and burnout amongst these individuals [15, 16]. However, before we consider such issues, we first provide a background to the RHC concept.

### RHC: Conceptual background and definitions

Broadly, ‘resilience’ is a term that has been referenced across academic and popular literature in many different ways; the term is used across a range of fields and disciplines, from psychiatry and the understanding of individual human responses to stress, to biology and understanding of resilience in organisms and ecological system functioning. However, the core features of resilience, regardless of the field or discipline, include the ability of the system to: sustain its operations despite stress, perturbations, and unforeseen events; recover from a major disruption; or adapt to new circumstances [17]. In sum, across fields and disciplines that reference resilience, there is an emphasis on the individuals’, communities’ or organisations’ ability to regain equilibrium in circumstances of changes, or to adapt to new norms, forms, and practices.

The first application of resilience to healthcare can be dated to around 2012 [18]. The concept was largely drawn from resilience engineering (i.e., where resilience is defined as a system’s capacity for flexibility, robustness, and adaptability in response to changing circumstances so that performance, including safety, is maintained; [19]) and disaster resilience (i.e., the application of resilience thinking in public health responses to major crises such as natural disasters and outbreaks of infectious diseases; [17]). At the time, RHC was defined by Hollnagel et al. [18] as the “ability of the healthcare system to adjust its functioning prior to, during, or following changes and disturbances, so that it can sustain required performance under both expected and unexpected conditions" (pxxv).

While Hollnagel’s definition underpinned the RHC theoretical framework, translation of the concept into practice was more problematic, and little guidance was provided. More recently, Wiig et al. [20] in their commencement of an international five-year program of research on RHC conducted at scale, reviewed the broader resilience literature (including ecology, engineering and psychology) to develop an operational definition of resilience to underpin the work. Drawing on the various concepts of resilience across multiple fields and disciplines, Wiig et al. [20] defined RHC as the capacity to consistently deliver safe, high-quality care through adaptations at *multiple system levels* in the face of challenges and disruptions. Importantly, this definition encompasses resilience-based efforts and analysis across system levels (micro, meso, macro). This definition of RHC was developed to be applicable regardless of the level of analysis or type of system component under investigation.

### RHC: Frameworks

At the foundation of RHC, Hollnagel et al. [18] proposed ‘four cornerstones of resilience’, which describe a system’s resilience in terms of how well it can: respond (knowing what to do and adjust to disturbances and changes), monitor (knowing what to look for and monitor what happens in and around the system), anticipate (knowing what to expect and prepare for) and learn (knowing what has happened and learn from experience). Hollnagel et al. [18] further suggested that these potentials are interactive and co-dependent, meaning that if the system fails in one of them, this will affect the other. Notably, these potentials have been used to operationalise resilience and serve as a framework for research that has been widely adopted in the analysis of RHC [10, 21].

In a review of the empirical studies of RHC over a ten year period, Berg and Aase [22] developed a complementary, theoretically and empirically driven framework for identifying key resilient concepts or characteristics across systems levels (individual: micro, team: meso, management/organization: macro) relating to: anticipation, sensemaking, adaption and tradeoffs [22]. Berg and Aase [22] defined *anticipation* as the ability to anticipate and prepare for hazards or changes before they occur. *Adaptations* refer to adjustments being made “as a result of coping with complexity, and the need to be flexible and improvise when necessary” [22]. *Sensemaking*, following the seminal work of Weick [23], is a meaning-making process by which individuals work to comprehend uncertain and unexpected events in order to adapt, and referred to as collective sensemaking when taking place as a team [22]. *Tradeoffs* relate to the act of decision-making and are represented as cognitive tradeoffs that individuals make between competing goals and tensions within teams [22]. Although there are some differences in how each of the characteristics are described at different system levels, they can be conceptualized in similar ways, allowing for common terminology for resilience at the individual, team and management levels [20, 22].

### The current study

We aimed to fill the gap in the limited research examining resilience across multiple system levels in a community-based setting. The objective of this study was to conduct a secondary analysis of interview data collected as part of a larger study [24] to examine resilient characteristics across varying system levels during the implementation of a planned large-scale community-based suicide prevention intervention known as ‘LifeSpan’.

The LifeSpan initiative is a multilevel community-wide suicide prevention intervention. It was implemented over a two-year period across four distinct geographical regions in Australia’s most populous state, New South Wales (NSW). The intervention included universal strategies designed to reach the entire population (regardless of risk), as well as selective strategies which targeted subgroups of the general population that were determined to be at risk for suicide and indicated strategies for individuals experiencing early signs of suicide crisis or behaviour. Each site (i.e., region) had its own local implementation team that was supported by a central implementation team and a research team based at the Black Dog Institute (BDI) [25].

This study focussed on increasing the understanding of resilience characteristics at different levels during implementation of LifeSpan across four diverse healthcare contexts. The specific study aims were to:

- (1) Identify characteristics of resilience during implementation across different system levels (individual, team, management).
- (2) Analyse both impediments to, and strategies that supported, resilient performance during the implementation of LifeSpan at different system levels.
- (3) Examine the individual consequences for LifeSpan personnel of maintaining resilient performance at system level.

## Method

### Study design

This study is based on a secondary thematic analysis of qualitative interview data collected during the implementation evaluation study of the LifeSpan initiative [24], using the theoretically and empirically driven Berg and Aase [22] framework to identify characteristics of resilience across multiple system levels. Secondary analysis of existing datasets has increasingly been considered an effective way of maximising knowledge and potential benefits of research [26]. Ethical approval for the study was granted by the Hunter New England Human Research Ethics Committee (2019/ETH03862). Participants provided consent for their data to be used for the implementation evaluation and for related projects. The consolidated criteria for reporting qualitative research (COREQ) checklist is shown in Supplementary File 1.

### The LifeSpan initiative

LifeSpan is a comprehensive and complex whole community suicide prevention program developed and supported by The Black Dog Institute (BDI), Australia’s medical research institute dedicated to researching and improving mental health for all ages [27]. LifeSpan is complex due to the multiple interacting components of the initiative and owing to the intricacies and myriad interactions across the various community services and settings into which the implementation is introduced [28]. The LifeSpan model includes the simultaneous implementation of nine suicide prevention strategies necessitating engagement across numerous sectors (e.g., health, mental health and community services; non-government organisations, local government, workplaces, schools) [27]. Details of the LifeSpan program and the evidence behind individual strategies are given elsewhere [25] but briefly, the nine strategies involve: (1) improving emergency and follow up care for those in suicidal crisis; (2) using evidence-based treatments; (3) better equipping primary care to identify and support people in distress; (4) improving the competency and confidence of frontline workers to deal with suicidal crisis; (5) partnering with schools to promote help-seeking, mental well-being and personal resilience; (6) engaging the community and providing opportunities to be part of the change; (7) training the community to recognise and respond to suicidality; (8) encouraging safe and purposeful media reporting; and (9) improving safety and reducing access to means of suicide [25, 29].

LifeSpan aimed to deliver the nine suicide prevention strategies simultaneously across four different regions. Regions were defined as being one or more Local Government Areas (LGAs) which interact meaningfully and fall within the boundaries of a single Local Health District (LHD) or Primary-Health Network (PHN). Lead agencies (LHDs, PHNs or non-government organisations) expressed interest in participating in the LifeSpan project and demonstrated that they had strong relationships with other key mental health agencies (e.g., EveryMind, https://everymind.org.au/; LifeLine https://www.lifeline.org.au/) with whom they would collaborate to deliver the nine strategies within the LifeSpan model. The LifeSpan project funding was distributed to the lead agency.

The delivery of the nine LifeSpan strategies was expected to be managed and implemented within each region by LifeSpan coordinators in collaboration with the LifeSpan management team at BDI. The LifeSpan management team was supported by several other BDI teams, including research and evaluation, data, and implementation teams. There was also a central collaborative group with participation of community members with lived experience, at each region that acted as a hub for program planning along with a number of smaller working groups that focused activity within each region.

### Study setting

LifeSpan was trialled in four geographical regions (henceforth, referred to as sites) across regional and rural NSW, Australia. The implementation evaluation research team from our research group at the Australian Institute of Health Innovation (AIHI), first engaged each site in mid-2019 in the final year of active implementation of the LifeSpan initiative. All personnel across each site were made aware of an external evaluation of the implementation of the Lifespan program and were invited to take part.

### Qualitative data collection procedure

Qualitative data were collected through semi-structured interviews and focus groups at each site. The researchers scheduled interviews with LifeSpan coordinators at each site and aligned focus groups to coincide with regular collaborative and smaller working group meetings to maximise participation. Interviews were also conducted with the BDI LifeSpan management team members and key personnel from the BDI research and evaluation, data, and implementation teams. A total of 53 individuals participated in individual interviews, small groups or focus groups across the sites involving stakeholders engaged in the implementation process. A semi-structured interview guide was developed in which questions acted as prompts allowing for the exploration of relevant issues as they emerged in both the face-to-face interviews and focus groups conversations [30]. Questions explored the fidelity of LifeSpan in each region, the barriers and enablers to implementation, insights into key roles associated with implementation, and the identification of strategies that facilitated the delivery of LifeSpan. All interviews and focus groups were conducted by three senior health services researchers (YZ, LAE, JCL), who are female and have extensive experience in qualitative research and were audio recorded and transcribed verbatim. The researchers had no formal pre-existing relationship prior to the study commencement. Interviews and focus groups lasted around an hour.

### Coding and data analysis

For this study, we examined the transcripts for the presence of resilient characteristics across varying system levels and sought to identify strategies used by the implementation teams to support resilience during the implementation of the LifeSpan intervention. Transcripts were imported into NVivo [31] for data management and analysis. We focused our analyses on eight transcripts involving thirteen key personnel involved in the day-to-day management and implementation of LifeSpan, including the nine coordinators from the four LifeSpan sites and four personnel from the central BDI management team. These transcripts were selected for this study as they involved key personnel involved in the day-to-day program implementation at each site and the project management team. These selected transcripts included four individual interviews and four small group interviews (each with 2-3 participants) with the key personnel, and did not include analysis of the focus group transcripts, involving the broader LifeSpan collaborative or working group members.

The eight transcripts were analysed using a directed content analysis approach [32] which included the use of deductive coding of characteristics of resilient performance according to Berg and Aase’s [21] framework and inductive coding to identify patterns driven by the data. Deductive coding of resilience characteristics (anticipation, adaptation, sensemaking, trade-offs) were classified at one of three system levels: individual (micro), group (meso) and management (macro) (see Table 1 for definitions as applied in this study).

**Table 1:** Deductive analytic framework as applied in this study

*Deductive codes*	*Definition*
Anticipation	Knowing what to expect or being able to foreshadow developments further into the future, such as potential issues or new opportunities.
Adaptation	Knowing what to do or being able to adjust to unexpected events in complex situations.
Sensemaking	A sense of, or shared understanding of, what is happening.
Trade-offs	When competing goals and tensions emerge individuals or teams bargain, negotiate and decide what to sacrifice.

This framework and definitions were drawn from work of Berg and Aase [22] and were summarised in this way for the purposes of this study.

Simultaneous inductive coding meant researchers also performed open coding and sought to adapt the framework where necessary and incorporate additional codes to identify resilience strategies and impediments to resilient performance. Four authors (LAE, CP, MS, JCL) double-coded the eight transcripts by different combinations (each author coded four transcripts). Three weekly meetings with the wider authorship team (LAE, CP, MS, JCL, RCW, YZ) to discuss the categorisation of codes and themes throughout the analysis process, identify discrepancies (with author, JB, available to resolve any disagreements), and ensure coding consensus and maximise rigour. Although inter-rater reliability was not formally assessed, the use of strong analytic framework and regular discussions, supported consensus and consistency in coding. Further, after all transcripts were coded, one author (LAE) iteratively read through all transcripts and codes to further ensure consistency of coding, discussing the process with the broader research team (LAE, CP, MS, JCL, RCW, ER, YZ, JB) at a final meeting. Through examination of codes and coded data, themes were developed that identify issues impacting resilient performance and resilient strategies identified across the system levels.

## Results

Within the transcripts, we found indicators of the four characteristics of resilience (anticipation, adaptation, sensemaking, trade-offs) across multiple system levels, thus providing support for the utility of the Berg and Aase [22] resilience framework. Representative quotes pertaining to each of the characteristics across system levels are presented in Table 2. Deductive coding revealed substantial interrelation between the four features. For example, sensemaking often occurred with adaptations (see Table 2).

**Table 2:** Deductive coding results with example quotations of four characteristics of resilience across system levels

*Resilience characteristic*	*Individual*	*Team*	*Management*
Anticipation	“[In anticipation of future needs]…one of the things that I did do when I first started was brainstorm a list of people - key stakeholders within the mental health and suicide sector on the [location], who I needed to go and talk to” (Site B, Coordinator 1)	“As things are growing… it's sort of prioritizing growing things, and thinking forward, but also keeping the current processes happening” (Site A, Coordinator 2)	“[BDI manager] fought very hard for it to be a [higher level] position [LifeSpan Coordinator] so that when you have to go and talk to other stakeholders, the title means that you’ve got the authority to then speak to them” (Site D, Coordinator 3)
Sensemaking	“I just felt like I was just a complete nag ringing up all the time, and just I had so many questions. But it was just trying to figure it out in my head, it had to click in my head, how we're going to structure this” (Site B, Coordinator 2) “I remember just not being entirely clear why so much effort was being put into writing these implementation guides…and actually sitting back watching how it was playing out with the BDI implementation team with the site coordinators and in hindsight I don’t think I recognised it all that clearly at the time and I actually think it did some damage to the relationships…” (BDI, Manager 1)	“there's so many things that we were trying to do that were hard, and if we didn't have the other person to bounce ideas off or go, “am I completely off?”. Honestly I think there were times we thought if we hadn't been able to talk to the other person you would think you were losing your mind because, kind of everything you've said, and then what happens, just don't marry up, so I think that that was helpful” (Site C, Coordinator 1)	“They [BDI management] are still trying to figure that out too and navigate those waters… they just didn't have that sort of thinking. They're coming from research, not so much from implementation. So this is kind of all new for them…So, I think that was one of their challenges...that they're still trying to figure out” (Site B, Coordinator 1)
Trade-offs	“The stuff that needed to happen was a small proportion of what I ever actually got to do, so that really threw a spanner in things in terms of how much work we're actually able to do, in reality…like in actually making this work” (Site C, Coordinator 2)	“We got to the stage, where there were lots of things we had to shelve because of capacity” (Site C, Coordinator 1) “Because it was so big you couldn't do everything so each site would kind of, you know, based on different coordinators interest you would, or based on interest from your local group, we’d progress some further than others” (Site D, Coordinator 2)	“Focusing on one or two things at a time, and trying to make sure stakeholders are engaged and it's implemented well is much more important… it's good to be ambitious to inspire hope, and for motivation, but you also need to be realistic about what can be achieved” (BDI Manager 3)
Adaptation	“I think you kind of have to think on your feet a little bit at times, and just go "We don't have the resources for that. So I'll just figure it out." And I'll just try and do, like, I've had to become a graphic designer. But I've just had to tap into skills I've had years ago. I'll try and knock something up because we don't have the funds to do that” (Site B, Coordinator 2)	“the thing that has made a difference is to maintain engagement. [Engagement is what] you need, if you're bringing something that people don't want, you need to be able to take on board the feedback and take back something new” (Site C, Coordinator 1)	“The other thing that we did and I'm still working on is rather than having a research manager, which is what we did have, we're now looking for a trial manager” (BDI, Manager 1) “the communication structures have changed quite dramatically, to try and address that issue” (BDI, Manager 2)

Through inductive coding, several themes were identified at the management and broader system level impeding the successful implementation of the LifeSpan initiative, which have been woven into the results with the characteristics and strategies of resilience below, and which provide an extension to the Berg and Aase [22] resilience framework (see Table 3 for a summary of the inductive thematic codes for impediments and strategies).

**Table 3:** Summary of inductive thematic codes

**Inductive codes**	**Description**	**Example**	**System level**
**Strategies**			
Creative use of resources	Individuals and teams displaying creative use of resources (e.g., equipment, networks).	“… put in some money, and ended up getting enough for two courses and face-to-face. So without that kind of coordination and collaboration around costing…that kind of opportunity just wouldn’t exist.” (Site C, Coordinator 2)	Individual, Team
Prioritise tasks/goals	Teams establishing priorities at the expense of others (i.e., making trade-offs) and being opportunistic.	“In terms of the nine strategies that were identified, [we] clustered them into three or four…that [made] it more manageable…things can be prioritised and started at different time points…we were running out of time, it was completely pragmatic” (Site C, Coordinator 1)	Team
External engagement and collaboration	Growing network with external individuals, groups and organisations, and engaging community and clinical champions.	“actually making time to meet with her regularly... And to just create that relationship” (Site B, Coordinator 2)	Team
Teambuilding and collaborative learning	Building relationships between teams and with management.	“so the next week, I started individual calls with each site and asked them like…what’s going on, what do you need…And then set up those calls so that they repeated weekly” (BDI Manager 3)	Management
Monitoring & feedback	Management quantitatively monitoring progress at each site and provide feedback.	“dashboards…with monitoring of how the sites were going” (Site D, Coordinator 2)	Management
**Impediments**			
Complexity	Complexity of the health system and complexity of the intervention.	“the scope of the project was so huge, and it covered so many different sectors and organisations” (Site C Coordinator 2)	Across
Misalignments in priorities/needs	Misalignments in priorities and needs between the team, management and broader system levels.	“Whereas, the things that were a priority to us, weren’t necessarily the same priority to BDI” (Site A, Coordinator 1)	Across
Inadequate resources	Deficiency of resources (money, staff, skill-mix) at the individual, team, management and broader system level.	“It wasn't sufficient to implement the whole nine strategies. It wasn't sufficient to evaluate and see whether they had an impact. It wasn't sufficient in all likelihood to actually reduce deaths.” (Site B, Coordinator 1)	Across
Staff turnover	Critical staff (including site coordinators and managers) and program and clinical champions leaving at critical times.	“I think one of the things that often happens in change programs is that key people leave at critical times. And [BDI Senior Manager] left. And she was a kind of mobilising force and enthusiastic and getting everyone excited…it certainly had an impact on me after that” (BDI Manager 2)	Across
**Consequences**			
Built personal and collective resilience	Ability to withstand, adapt to, and recover from adversity, at the individual and team level.	“Because as much as it’s a shift for us, I know it’s a shift for them as well. And it’s just been a really painful process, because we’ve all had to go through this shifting together” (BDI Manager 4)	Individual. team
Stress and burnout	Workplace stress and exhaustion caused by feeling overwhelmed and emotionally drained.	“we were burnt out…you lose momentum” (Site A, Coordinator 2)	Individual

Notably what was apparent was that impediments to resilience at one system level may shape resilience at other levels. Firstly, participants identified that the sheer *complexity* of the LifeSpan model, which consisted of nine strategies, and “covering so many different sectors and organisations”, was particularly challenging:

“You cannot do a trial of such a complex systems approach and a community led approach in two and a half years. It’s just not doable.” (Site A, Coordinator 1)

Collectively, BDI managers and site coordinators expressed a sense of being overwhelmed with the sheer scale of the work ahead of them:

“I think the overwhelming sense was how the hell are we actually going to do this. It was just chaos and every time we thought about it, all of us felt completely overwhelmed and I think, almost, unable to imagine what it was going to look like in some ways” (BDI Manager 1)

Due to the complexity and scale of the work, LifeSpan coordinating teams at each site had to *prioritise tasks/goals* and choose strategies that would provide the “biggest bang for buck”:

“the actual expectations on [the Site Coordinators] to be getting a number of things up and running at the same…it was too big too fast.” (BDI Manager 1)

Site coordinators soon recognised that the BDI management team were not the knowledge brokers that they had anticipated them to be (i.e., sensemaking), which prompted the need for learning and tackling problems at a site level, partly due to *misalignments in priorities/needs* between BDI management and the sites:

“I wanted more from [the] Black Dog [Institute] as an expert knowledge holder or knowledge provider. But in a lot of ways, they were learning as much as we were…when we were tackling problems…they didn’t necessarily have any more wisdom than we had. And I think because we were the ones facing it, we were the ones having to sort it out…there was more pressure. We had to make a choice or had to work something out. Whereas, the things that were a priority to us, weren’t necessarily the same priority to BDI” (Site C, Coordinator 1)

As a result, LifeSpan coordinating teams at each site had to quickly learn, prioritise and make trade-offs between competing goals due to misalignments between demands from BDI management team and the time available and expectations to implement the complex strategies:

“*So a lot of the way our timing of stuff happened was, whatever was ready was what was rolled out, which, because we're running out of time, it was completely pragmatic.”* (Site A, Coordinator 1)

With localised sensemaking and adaptations occurring at each site, this led to significant variations in what was being implemented and how. As aphoristically put by one BDI manager, it was largely about the sites being “opportunistic” and “playing to their strengths”:

“So each of the sites has played to their strengths or there’s been some kind of opportunity that’s come up, which meant that the ability to engage the Stakeholders has been easier…the sites are pretty good at grabbing those opportunities and making the most of them.” (BDI Manager 1)

*Inadequate resources* were also identified as an impediment to resilience:

“it was a very ambitious project with very little resources really, very little money, for what they wanted to achieve.” (Site B, Coordinator 2)

Although the BDI was leading the intervention trial, the project funding for sites was held and controlled by the lead agency (PHNs and/or LHDs). This caused significant issues for some of the sites in accessing resources. One lead agency withheld access to funding for project related activities:

“[The lead agency] hasn’t been approving things. They’re not fully across the budget so they just assume there’s not a lot of funds. Like, it’s just that level of disconnect, and slow bureaucracy and the priorities are elsewhere.” (BDI Manager 3)

As a result, at each of the sites, coordinating teams had to “make better use of resources” available to them (i.e., creative use of resources), as well as flex and adapt to identify additional sources of support.

*Staff turnover* at an individual, team and management level also hindered stability and continuity of the implementation process, thereby creating challenges at an individual level:

“So for me personally...the change of staff was quite challenging.” (Site D, Coordinator 3)

Change in senior leadership at BDI and the lead agencies (LHDs and PHNs) also meant that new managers came into the program after others had already put wheels in motion:

“[The lead agency], the people who put the initial expression of interest in are no longer involved for a variety of reasons…And so it’s been quite a challenge.” (Site B, Coordinator 1)

Staff turnover had an obvious impact on team morale, and as a result the BDI management team put a lot of effort into *teambuilding* and *collaborative learning*. A strategy the management team adopted was to run regular ‘SIT-INS’. These were essentially co-design workshops where the coordinators from each of the four sites would meet together with the BDI management team. These workshops allowed everyone to “get on the same page”, anticipate and sensemake as a cohesive team, or as one manager aptly said “unpack things together”.

“That’s how the SIT-IN was born…this was seen as a shortcut to get a little bit ahead…so we can just get through all these things [together]” (BDI Manager 4)

Another issue highlighted was that several of the BDI managers and site coordinators lacked the necessary skills and experience in implementing complex interventions in the community and health system (i.e., inadequate resources). When developing implementation plans, one BDI manager described the process as “developing plans as we went along, on the fly”. Another said:

“The scale and the levels at which we’re operating meant that, you know, as we’re trying to pave the road just ahead of everything happening, this does not allow a lot of time to kind of get that right, it was a mismatch.” (BDI Manager 3)

The BDI management team co-developed implementation guides with the site coordinators at the SIT-IN meetings. Although the guides were initially useful for BDI “to actually work out what we were doing” and for the sites so that “everyone then became intimately across it”, ultimately, they highlighted the misalignment between the needs of the BDI management team and the needs of each of the site coordinating teams (i.e., misalignments in priorities/needs):

“it doesn’t have the context I need…they are very research focused rather than implementation focused…they would spend entire SIT meetings going through the implementation guides…and how they are going to put the research into [an academic] paper, but that does not matter to the people on the ground.” (Site D, Coordinator 2)

Some BDI managers recognised this misalignment and adapted the purpose of the SIT-IN meetings and sought to “connect” with each of the sites and “get some honest feedback to build stronger relationships” by making regular fortnightly calls to the site coordinators.

This new approach from BDI management was well received by the site coordinators:

“[BDI manager] will ring up maybe once every couple of weeks and just check in and say “how’s everything going?” “Are you ok?”…[BDI manager’s] like “Look I know how to do…I’ll sort that out for you”…we couldn’t have done it without [BDI manager]” (Site C, Coordinator 2)

Another strategy adopted from the BDI management team was to quantitatively *monitor progress* at each of the sites, and *feedback* that information to their respective coordinating teams:

“collect data to see whether it works and then feed that back to the sites. We work with the sites and make sure that they are timely data on what implementation strategies are working and which ones aren’t, and then revise. So a constant cycle of testing, getting evidence, testing…” (BDI Manager 1)

A main theme voiced across transcripts was that local community networks and relationships had a beneficial impact in cultivating resilience. Site coordinators who had existing local relationships and networks were able to call on them for support and involvement (i.e., creative use of resources):

“So there were a few pre-existing relationships. And so some of the sites that I knew back then, were able to be engaged as well” (Site C, Coordinator 1)

However, growing a network with individuals, teams and external organisations was considered key to success (i.e., *external engagement and collaboration*):

“Developing relationships with people is the only way in… If you can't have positive relationships with people, you won't get anywhere.” (Site B, Coordinator 1)

Another strategy employed by the site coordinating teams was to engage external community and clinician “champions”. These champions served as role models for the LifeSpan initiative, increasing knowledge of, and participation in, LifeSpan across the community.

“our enablers have been our Champions, and we’ve nurtured those…she did the [Question, Persuade, Refer Suicide Prevention Training] and then she did the Championship training and she’s still championing us. We’ve got a few people like that and they’re worth their weight in gold.” (Site C, Coordinator 1)

Ultimately, being able to encourage meaningful engagement and collaboration between the community, local health services and non-government organisations was highlighted as pivotal:

“Ok so the relationships, so I’m most proud of that. We actually did bring people together, we did strengthen relations and people value that.” (Site A, Coordinator 2)

Finally, what was clear from the interviews was that the personnel at the sites and at BDI were philosophically and emotionally invested in the LifeSpan initiative. However, dealing with the complexity of the intervention, and the need to constantly learn and make adaptations in response to unexpected variation and changes, came at a personal cost to those most involved. A number of the site coordinators reported that, at an individual level, they were emotionally exhausted from trying to make the initiative a success:

“really frustrating and tiring and some days you are not really up for it.” (Site A, Coordinator 1)

BDI managers were no different, reporting ongoing stress and burnout throughout the project. However, upon reflection, one BDI manager identified that they have *built personal resilience* as a result of the challenges endured:

“everything you could probably think could go wrong in like any professional environment, I got to experience it in like three years. And nothing can surprise me anymore on a personal level. And so it’s been kind of like this really heavy resilience training that I’ve just gone through.” (BDI Manager 3)

Working collaboratively created a *collective resilience* among staff members at the sites and at the BDI:

“They were finding their way too, like we were all learning together…I had the mindset that we are all in this together.” (Site B, Coordinator 2)

## Discussion

We designed this study as a key step in advancing our understanding of the characteristics of resilience at various system levels outside of the clinical ‘sharp end’ of hospital care. Here, the LifeSpan initiative’s implementation can be broadly considered the ‘trigger’ that activated capacities for resilience within the system (the “resilient to what?”; [20]), though there were clearly many threats and obstacles to enacting resilience throughout the project. The complexity of the multi-modal intervention, inadequate resources, staff turnover, lack of skills and experience, incompatible goals and priorities between system levels, as well as incompatible local governance structures, were some of the key identified impediments.

The system levels in this study comprised the individual, team and management levels, though it was clear from the results that the presentations of resilience at each level of analysis were not discrete, with a high degree of interconnectivity between the various system levels. For example, from the quotes it appeared that sensemaking often occurred with adaptations. Therefore, the results of this study provide empirical underpinnings to those theorists who have conceptualised system resilience as a “multi-level phenomenon” (e.g., [33]).

Despite advances in resilience research in recent years, most studies within healthcare neglect to consider that individuals are embedded within teams, and teams are embedded within organisations and their broader systems [34]; despite the broader organizational resilience literature adopting this view [35, 36]. However, in taking a multilevel systems perspective, we argue that a good first step is to adopt a resilience framework for research design and analysis. Although the resilience characteristics identified by Berg and Aase [22] were conceptualised somewhat differently between system levels, the framework as applied here proved to have high utility, allowing for common terminology for resilience characteristics to be mobilised across system levels during the implementation of a planned system innovation. An additional benefit is that the framework proved applicable outside of the clinical microsystems in hospitals to a broader community mental health system of care. The results from this study also extend this model in the identification of strategies and impediments to resilient performance.

Perhaps most importantly, the study suggests that resilience at one system level may shape resilience at other levels in complex ways. For example, in times when there was inflexible management at the executive and upper management levels from BDI and the LHDs/PHNs, individuals and teams at the frontlines of care were pushed to flex, respond and adapt accordingly. Additionally, our results are concordant with the view of Caza et al.’s [33] that there can be a “dark side or downside of resilience” (p.346). What started out as resilient work practices, over time, led to stress and burnout in a number of the personnel most closely involved in LifeSpan. These findings are particularly applicable in light of the COVID-19 pandemic, which has caused major disruption across all system levels and resulted in widespread mental health issues and burnout amongst frontline healthcare workers [15, 16].

While previous studies of resilience have typically solely focused on the ‘benefits’ of resilience to the system (e.g., performance, efficiency, safety outcomes; [33]), the results here point to the need to consider the possibility for negative impacts as well (e.g., by including measures of stress, job satisfaction and burnout). Caza et al. [33] have also pointed to additional negative impacts at other system levels that should also be considered, including inefficiency and organizational rigidity. With this, there is also the need to collect data longitudinally to increase our understanding of causal processes between the various system levels. In this type of analysis, quantitative approaches may facilitate a relatively objective comparison of changes over time. However, for investigating complex processes and how they evolved over time, we suggest that in-depth qualitative approaches may be best.

Although there were notable downsides and many challenges experienced along the way, BDI managers enacted several strategies to support resilient performance, including teambuilding, collaborative learning, building relationships, monitoring progress and providing feedback. They sought to plan for upcoming changes and improve communication channels, and shifted tack when things were not working as planned. Likewise, at the site coordinator team level, several strategies were employed to enhance system functioning, including the creative use of resources, finding additional sources of support, drawing on and growing community networks and relationships, and engaging with external community and clinical champions. It could be argued that all these strategies promoted possibilities for learning, growth and development within the broader LifeSpan team, and ultimately may have enhanced healthcare system functioning within each of the sites involved [37]. Indeed, as identified from our social network survey study of LifeSpan [24], the site coordinators were empirically identified as the “key players in the networks”, and “were noted to be exceptional people who magnified the benefits of collaboration” (p.1). All in all, the efforts of the LifeSpan coordinators in building a collaborative network in each site was identified as a key success factor for the implementation of LifeSpan [24].

**Fig. 1:**
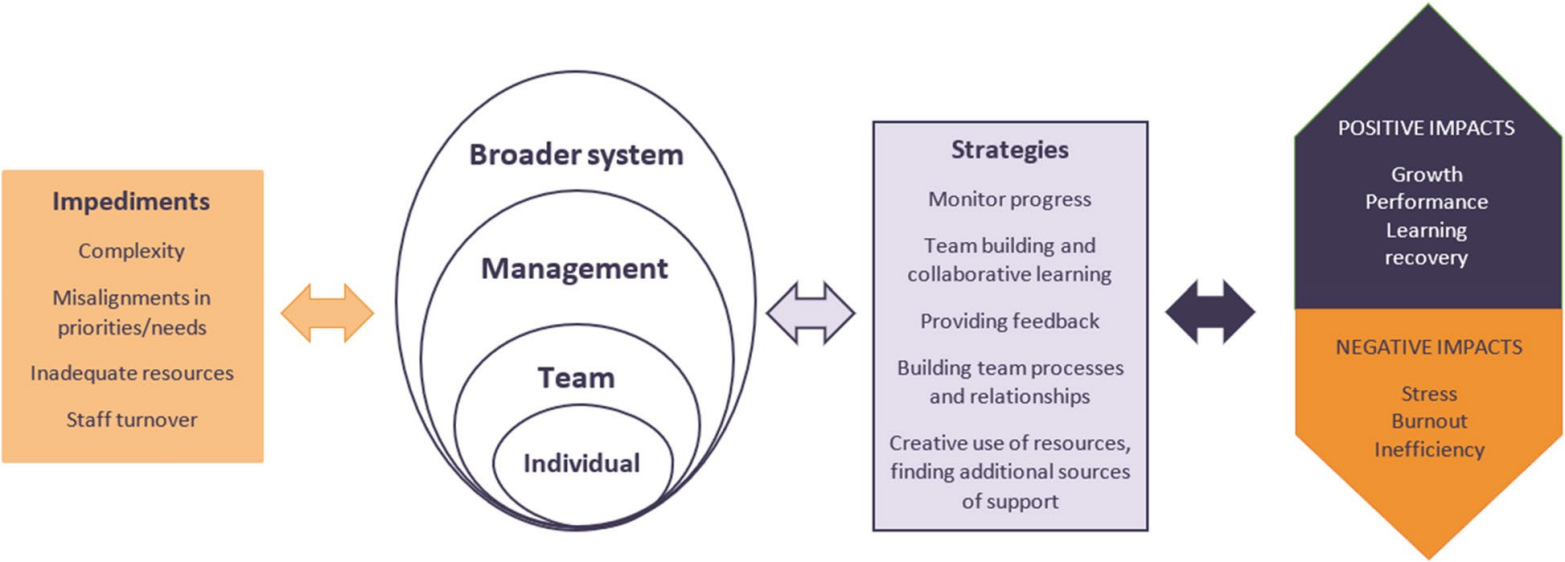
Multilevel model of systems resilience

Many of the strategies identified from this study here share similarities with the “capacities for resilient performance” identified from a variety of RHC research projects across different contexts and levels being conducted by researchers from SHARE, the Centre for Resilience in Healthcare in Norway [9, 37, 38] and the Australian Institute of Health Innovation in Australia [10, 39, 40]. This study thereby contributes to this work with key insights for intervention development and scoping of potential adaptable strategies that can be employed to enhance system functioning, especially during implementation. Understanding factors that develop or enhance RHC is critical to developing interventions and tools for strengthening their resilience [41]. Further, based on the literature, our related work [9, 10, 37–40] and the results of this study, we have proposed a visual multilevel model of systems resilience (Figure 1) to contribute to this work and to assist with future research design and analysis.

## Limitations

The LifeSpan interviews were not originally designed as a resilience project, and thus the interview questions were not tailored specifically with resilience in mind, however, the data clearly included rich data on how personnel working at various levels were constantly sensemaking and making adaptations in the face of risks and challenges. The extent of the challenges they faced, and their adaptive responses, triggered our conceptualisation and development of adopting a resilience lens for this study. Another limitation was the restricted focus on individual, team and management levels; a more comprehensive multi-level approach could have also included the broader community, government and policy levels.

## Conclusions

As Caza et al. (2020) neatly said the “multi-level messiness of resilience” suggests that we “need to step back from a single-level perspective on resilience, taking a much broader view in order to understand how it emerges over time” (p.345). Operationalising resilience from a multilevel perspective, examining both positive and negative impacts, as well as expanding the scope of the data collected (over time, across levels), is needed to advance the RHC field forward so that we can develop a richer, more nuanced understanding of resilience.

## Supplementary Information

**Additional file 1.**

## Data Availability

The data analysed during the current study are not publicly available due to ethical restrictions, but are available from the corresponding author on reasonable request and with appropriate ethics approval.

## Abbreviations

AIHI: Australian Institute of Health Innovation
BDI: Black Dog Institute
LHD: Local Health District
NSW: New South Wales
PHN: Primary Health Network
RHC: Resilient Health Care
WAD: Work as Done
WAI: Work as Imagined
