# Supplementary material for: Systems resilience in the implementation of a large-scale suicide prevention intervention: a qualitative study using a multilevel theoretical approach
Source: BMC Health Serv Res. 2023 Jul 11;23:745. doi: 10.1186/s12913-023-09769-x (PMC10334581; doi:10.1186/s12913-023-09769-x)
Supplement: Supplementary file 1 — Additional file 1. [file 12913_2023_9769_MOESM1_ESM.docx]

**COREQ Checklist**

| **No** | **Item** | **Guide Questions/Description** | **Reported on Page No. and Section** |
| --- | --- | --- | --- |
| Domain 1: Research team and reflexivity | | | |
| Personal characteristics | | | |
|  | Interviewer/facilitator | Which author/s conducted the interview or focus group? | Page 9, Method, Qualitative data collection procedure. |
|  | Credentials | What were the researcher's credentials? E.g. PhD, MD | Page 9, Method, Qualitative data collection procedure.  Additional information: All three researchers have a PhD. |
|  | Occupation | What was their occupation at the time of the study? | Page 9, Method, Qualitative data collection procedure.  Additional information: title page. |
|  | Gender | Was the researcher male or female? | Page 9, Method, Qualitative data collection procedure. |
|  | Experience and training | What experience or training did the researcher have? | Page 9, Method, Qualitative data collection procedure.  Additional information: All three researchers have undertaken semi-structured interviews and focus groups over the past 10-15 years for the purposes of research and evaluation. They have authored numerous peer-reviewed papers reporting on qualitative analysis. |
| Relationship with participants | | | |
|  | Relationship established | Was a relationship established prior to study commencement? | Page 9, Method, Qualitative data collection procedure.  Additional information: The researchers met some of the participants at an introductory project meeting, but had not prior relationship prior to the study commencement. |
|  | Participant knowledge of the interviewer | What did the participants know about the researcher? e.g. personal goals, reasons for doing the research | Yes, at an introductory meeting, YZ and LAE briefly presented the reasons why the research was being conducted and its goals.  The participants information form that was provided was explicit in its description of the purpose of the research. |
|  | Interviewer characteristics | What characteristics were reported about the interviewer/facilitator? e.g. Bias, assumptions, reasons and interests in the research topic | At the introductory meeting, the research was framed as an implementation evaluation study of the LifeSpan initiative. |
| Domain 2: study design | | | |
| Theoretical framework | | | |
|  | Methodological orientation and Theory | What methodological orientation was stated to underpin the study? e.g. grounded theory, discourse analysis, ethnography, phenomenology, content analysis | Page 10, Method, Coding and data analysis, paragraph 2 |
| Participant selection | | | |
|  | Sampling | How were participants selected? e.g. purposive, convenience, consecutive, snowball | Page 8, Method, Study setting |
|  | Method of approach | How were participants approached? e.g. face-to-face, telephone, mail, email | Page 8, Method, Study Setting |
|  | Sample size | How many participants were in the study? | Page 9, Method, Qualitative data collection procedure.  Page 9, Method, Coding and data analysis, paragraph 1. |
|  | Non-participation | How many people refused to participate or dropped out? Reasons? | N/A |
| Setting | | | |
|  | Setting of data collection | Where was the data collected? e.g. home, clinic, workplace | Page 9, Method, Qualitative data collection procedure |
|  | Presence of non-participants | Was anyone else present besides the participants and researchers? | N/A |
|  | Description of sample | What are the important characteristics of the sample? e.g. demographic data, date | Page 9, Method, Qualitative data collection procedure |
| Data collection | | | |
|  | Interview guide | Were questions, prompts, guides provided by the authors? Was it pilot tested? | Page 9, Method, Qualitative data collection procedure |
|  | Repeat interviews | Were repeat interviews carried out? If yes, how many? | N/A |
|  | Audio/visual recording | Did the research use audio or visual recording to collect the data? | Page 9, Method, Qualitative data collection procedure |
|  | Field notes | Were field notes made during and/or after the interview or focus group? | N/A |
|  | Duration | What was the duration of the interviews or focus group? | Page 9, Method, Qualitative data collection procedure |
|  | Data saturation | Was data saturation discussed? | N/A |
|  | Transcripts returned | Were transcripts returned to participants for comment and/or correction? | No |
| Domain 3: analysis and findings | | | |
| Data analysis | | | |
|  | Number of data coders | How many data coders coded the data? | Page 10, Method, Coding and data analysis, paragraph 3 |
|  | Description of the coding tree | Did authors provide a description of the coding tree? | Page 10, Method, Coding and data analysis, paragraph 2 |
|  | Derivation of themes | Were themes identified in advance or derived from the data? | Page 10, Method and data analysis, paragraph 3 |
|  | Software | What software, if applicable, was used to manage the data? | Page 9, Method, Coding and data analysis, paragraph 1 |
|  | Participant checking | Did participants provide feedback on the findings? | No |
| Reporting | | | |
|  | Quotations presented | Were participant quotations presented to illustrate the themes / findings? Was each quotation identified? e.g. participant number | Page 11, Results, lines 251-253, lines 256-259, lines 262-265.  Page 12, Results, lines 270-277, lines 281-284, lines 288-291, lines 293-294, lines 299-302.  Page 13, Results lines 305-309, lines 312-313, lines 316-218, lines 319-322, lines 330-332.  Page 14, Results, lines 337-340, lines 346-350, lines 354-356, lines 358-361.  Page 15, Results, lines 365-369, lines 373-374, lines 377-379, lines 383-387, lines 391-393.  Page 16, Results, lines 400-401, line 402, lines 406-410, lines 413-417. |
|  | Data and findings consistent | Was there consistency between the data presented and the findings? | Page 17, Discussion, paragraphs 2 and 4.  Page 18, Discussion, paragraph 5. |
|  | Clarity of major themes | Were major themes clearly presented in the findings? | Page 11, Results, line 248.  Page 12, Results, line 268, line 292.  Page 13, Results, line 310.  Page 14, Results, line 333, line 344.  Page 15, Results, line 370, line 381. |
|  | Clarity of minor themes | Is there a description of diverse cases or discussion of minor themes? | Page 18, Discussion, paragraph 6. |
